# Depression and Obsessive-Compulsive Disorders Amid the COVID-19 Pandemic in Saudi Arabia

**DOI:** 10.7759/cureus.12978

**Published:** 2021-01-28

**Authors:** Noara AlHusseini, Muhammad Sajid, Afaf Altayeb, Shahd Alyousof, Haifa Alsheikh, Abdulrahman Alqahtani, Afrah Alsomali

**Affiliations:** 1 Epidemiology and Public Health, Alfaisal University College of Medicine, Riyadh, SAU; 2 Pathology, Alfaisal University College of Medicine, Riyadh, SAU; 3 Medicine, Alfaisal University College of Medicine, Riyadh, SAU; 4 Public Health Sciences, Alfaisal University College of Medicine, Riyadh, SAU

**Keywords:** covid-19, quarantine, mental health, depression, obsessive compulsive disorders

## Abstract

Introduction: Coronavirus disease 2019 (COVID-19) is a global pandemic with significant morbidity and mortality. The Saudi government adopted mandatory home quarantine and curfew hours for all residents, excluding essential service workers. During the lockdown, the public's fear of infection can adversely impact mental health, causing psychological distress. The objective of this research is to assess frequency of depression and obsessive-compulsive disorder (OCD) among the general population during COVID-19 pandemic in Saudi Arabia.

Methodology: This was a cross-sectional study using the Patient Health Questionnaire (PHQ-9) and Obsessive-Compulsive Inventory-Revised assessment test (OCI-R) in Arabic and English languages distributed via social media platforms. Chi-square test was used with significance determined at p<0.005.

Results: We received a total of 2187 responses. Our results showed that being female, single, and unemployed had a higher frequency of suffering from depression whereas higher income levels and higher education was associated with less depression frequency. Increasing age, males, married individuals, higher income groups, higher levels of education and employed individuals were more likely to have OCD during COVID-19 pandemic.

Conclusion: The COVID-19 pandemic period is associated with significant mental health risks among the Saudi population. The results can provide further scientific support to better understand the impact of quarantine on psychological distress and mental health during emergency and disaster situations.

## Introduction

Coronavirus disease 2019 (COVID-19) is derived from the coronavirus family. The virus first appeared in China, then continued to spread globally, and was declared a global pandemic by the World Health Organization (WHO) in March 2020 [1,2]. The most common symptoms of COVID-19 include fever, dry cough, shortness of breath, tiredness, headaches, nasal congestion, sore throat, diarrhea, and altered taste and smell sensations [2,3]. To minimize the virus transmission in Saudi Arabia, the government advised social distancing, implemented self-isolation for those affected, and adopted mandatory home-quarantine (lockdown) for all residents [4]. Public health measures to combat the virus's spread impacted daily activities, affecting jobs and causing financial insecurities. The lockdown situation is likely to have an adverse impact on mental health and wellbeing, leading to disruption in people’s daily routines [4-6]. Lockdown and quarantine can increase psychological distress leading to conditions such as anxiety, panic attacks, depression, post-traumatic stress disorder (PTSD), and obsessive-compulsive disorder (OCD) [4-6].

Depression is a mood disorder affecting different aspects of a person's life causing a decline in social and occupational functioning. It can also cause physical problems and decrease the person's ability to function in routine activities [7]. The severity of symptoms varies, from feelings of sadness to anxiety or restlessness, loss of interest, sleep disturbances, reduced appetite, and fatigue [8]. Social isolation and distancing can disrupt routines and create fear and anxiety, especially among those with mental health problems [4].

Obsessive-compulsive disorder is unwanted recurring obsessions (thoughts) and compulsions (behaviors) interfering with a person's daily tasks. Some of the common obsessions include fear of germs and wanting things in a symmetrical and orderly manner [9]. Compulsions include excessive cleaning and hand washing, constant checking and rechecking, and arranging things in a specific and precise way. The most common practice of OCD is fear of contamination [10]. OCD symptoms vary between individuals. However, people with OCD cannot control thoughts or behaviors and do not enjoy themselves, which affects daily living [9]. Nonetheless, recommended behaviors that prevent the spread of COVID-19, such as repeated hand washing, resemble some compulsive behaviors of OCD [10]. A study showed that OCD symptoms could worsen, as shown in a case of a patient who was previously in remission of OCD but relapsed with the spread of coronavirus [11].

The effect of COVID-19 lockdowns on mental illness frequency needs to be assessed to understand whether the lockdown will alter mental wellbeing in healthy individuals [12]. There is a lack of published data addressing the psychological impact of COVID-19, unlike severe acute respiratory syndrome (SARS) and Middle East respiratory syndrome (MERS) [12], which showed that one of the consequences is increased psychiatric morbidity, ranging from fear and PTSD to depression and anxiety [13-15]. Some recent studies have shown similar psychological distress patterns to SARS and MERS [16]. This study aimed to assess the frequency of depression and OCD among the general population in Saudi Arabia during the COVID-19 pandemic. 

## Materials and methods

The study targeted adults 18 years of age and above living in Saudi Arabia. With an estimated Saudi population of 33.7 million in 2020, the sample size was 384 with a 95% confidence level. Ethical approval was taken from Alfaisal University Institutional Review Board (IRB) (#20042), and all ethical requirements of the IRB were fulfilled during this study. 

The study was performed in the time of COVID-19 pandemic lockdowns. Therefore, no paper questionnaire was used, instead, only online surveys were distributed through social media platforms such as WhatsApp, Twitter, Instagram, Facebook, and LinkedIn. The questionnaire was made via Google Forms and was written in English and Arabic. Participation was voluntary and anonymous to ensure confidentiality and anonymity, completion of the questionnaire was construed as consent, and finally, access to the responses was limited to the authors only.

The questionnaire consisted of three main sections; first, demographic information such as age, gender, nationality, marital status, level of education, and employment status. Second, the Patient Health Questionnaire (PHQ-9) and finally, Obsessive-Compulsive Inventory-Revised (OCI-R) assessment test [17-19]. An Arabic language expert performed face validity for the Arabic version of the questionnaire. 

The PHQ-9, also known as Patient Depression Questionnaire, is a quick tool to assess depression among participants. The questionnaire is designed to cover social, occupational, or other important functioning areas [17,18]. The questionnaire is included in Appendix 1. The individual responses are calculated and interpreted as a total score as follows: 1-4 no or minimal depression, 5-9 mild depression, 10-14 moderate depression, 15-19 moderately severe depression, and 20-27 severe depression.

The OCI-R is a short version of the OCD assessment survey for assessing symptoms of OCD [19]. It consists of 18 questions that a person answers on a 5-point Likert scale. Scores are generated by adding the individual item scores. The possible range of scores is 0-72. Mean score for persons with OCD is 28.0 (SD = 13.53). The recommended cutoff score is 21, with scores at or above this level indicating the likely presence of OCD [20]. We analyzed the data via the jamovi project statistics analysis package (Sydney, Australia). Primary data points included age, gender, occupation, and socioeconomic levels. The Chi-square test was employed on categorical variables to examine associations. We determined significance when the p-value was <0.05.

## Results

We analyzed a total of 2186 responses. The socio-demographic characteristics of the respondents are shown in Table 1.

**Table 1:** Demographics of Respondents (N=2186) OCI-R: obsessive-compulsive inventory-revised assessment test

N=2186	n	%
Age Group (years)		
18-24	484	22.1%
25-35	620	28.4%
36-45	479	21.9%
46-55	348	15.9%
>55	255	11.7%
Gender		
Male	864	39.5%
Female	1322	60.5%
Nationality		
Saudi	1930	88.3%
Non-Saudi	256	11.7%
Marital status		
Single	834	38.2%
Married	1233	56.4%
Divorced	98	4.5 %
Widowed	21	1.0 %
Monthly income (SR)		
9,999 or less	563	25.8%
10,000-19,999	538	24.6%
20,000 or more	481	22.0%
Prefer not to answer	604	27.6%
Level of education		
High school, diploma or less	563	25.8 %
Bachelor’s degree	1191	54.5 %
Graduate degree	432	19.8 %
Employment status		
Employed	1225	56.0 %
Unemployed	961	44.0 %
Employment type		
Healthcare	286	23.4 %
Food industry, Supermarket, Delivery	52	4.3 %
Military force, Policemen	53	4.3 %
Other	829	68.0 %
How many times did you leave your home in last one week		
0	492	22.5 %
1-2 times/week	809	37.0 %
3-4 times/week	404	18.5 %
5-7 times/week	481	22.0 %
Diagnosed or direct contact with COVID19 case		
Yes, I have been diagnosed with No direct contact with a case	33	1.5 %
Yes, I have been diagnosed with direct contact with a confirmed case	27	1.2 %
No, I have not been diagnosed with No direct contact with a case	1888	86.4 %
No, I have not been diagnosed, but I had direct contact with a confirmed case	238	10.9 %
Depression calculated using the Patient Health Questionnaire (PHQ-9)		
No depression/ Minimal Depression	761	34.8 %
Mild Depression	667	30.5 %
Moderate Depression	407	18.6 %
Moderately Severe Depression	261	11.9 %
Severe Depression	90	4.1 %
OCD (OCI-R)		
Most likely has OCD	1365	62.4 %
Most likely does not have OCD	821	37.6 %

The results showed that 65.2% of the respondents had depression and 62.4% most likely had OCD. Analysis of categorical variables in relation to depression and OCD are shown in Table 2 and Table 3, respectively. 

**Table 2:** Chi-square test for Depression and Demographics

Depression	Minimal Depression			Mild Depression		Moderate Depression		Moderately Severe Depression		Severe Depression		P-value Chi-square test
Age (years)												
18-24	86	17.8%	139		28.7%	119	24.6%	105	21.7	35	7.2	< .001
25-35	160	25.8%	189		30.5%	140	22.6%	93	15.0%	38	6.1	
36-45	166	34.7%	180		37.6%	82	17.1%	42	8.8%	9	1.9%	
46-55	178	51.1%	98		28.2%	53	15.2%	11	3.2%	8	2.3%	
55+	171	67.1%	61		23.9%	13	5.1%	10	3.9%	0	0.0%	
Gender												
Male	387	44.8%	251		29.1%	132	15.3%	65	7.5%	29	3.4%	< .001
Female	374	28.3%	416		31.5%	275	20.8%	196	14.8%	61	4.6%	
Nationality												
Saudi	674	34.9%	590		30.6%	355	18.4%	231	12.0%	80	4.1%	.967
Non-Saudi	87	34.0%	77		30.1%	52	20.3%	30	11.7%	10	3.9%	
Marital Status												
Single	169	20.3%	232		27.8%	210	25.2%	171	20.5%	52	6.2%	<0.001
Married	545	44.2%	396		33.1%	182	18.4%	79	6.4%	31	2.5%	
Divorced	32	32.7%	35		35.7%	14	14.3%	10	10.2%	7	7.1%	
Widowed	15	71.4%	4		19.0%	1	4.8%	1	4.8%	0	0.0%	
Monthly Income												
0 to 9,999 SAR	152	27.0%	161		28.6%	126	22.4%	93	16.5%	31	5.5%	< .001
10,000 to 19,999 SAR	186	34.6%	165		30.7%	117	21.7%	49	9.1%	21	3.9%	
20,000 SAR or more	224	46.6%	157		32.6%	67	13.9%	25	5.2%	8	1.7%	
Prefer not to say	199	32.9%	184		30.5%	97	16.1%	94	15.6%	30	5.0%	
Education												
high school, Diploma or less	172	30.6%	156		27.7%	111	19.7%	94	16.7%	30	5.3%	< .001
Bachelor’s degree	409	34.3%	391		32.8%	212	17.8%	130	10.9%	49	4.1%	
Graduate degree	180	41.7%	120		27.8%	84	19.4%	37	8.6%	11	2.5%	
Employment												
Employed	446	36.4%	403		32.9%	222	18.1%	117	9.6%	37	3 %	< .001>
Unemployed	315	32.8%	264		27.5%	185	19.3%	144	15%	5	5.5%	

**Table 3:** Chi-square test for OCD and demographics

OCD	Most likely has OCD		P-value
	N	%	
Age			
18-24	233	48.1	<0.001
25-35	355	57.3	
36-45	320	66.8	
46-55	250	71.8	
55+	207	81.2	
Gender			
Male	582	67.4	<0.001
Female	783	59.2	
Marital Status			
Single	425	51	<0.001
Married	861	69.8	
Divorced	66	67.3	
Widowed	13	61.9	
Monthly Income			
0 to 9,999 SAR	320	56.8	<0.001
10,000 to 19,999 SAR	347	64.5	
20,000 SAR or more	354	73.6	
Preferer not to say	344	57	
Education			
High school, Diploma or less	309	54.9	<0.001
Bachelor’s degree	772	64.8	
Graduate degree	284	65.7	
Employment			
Employed	798	65.1	0.003
Unemployed	567	59	

The frequency of moderate to severe depression was more in females (40%) as compared to males (26%) (p<0.001). The frequency of severe depression was highest among the youngest age group 18-24 years (7.2%) (p<0.01). Single individuals (52%) were more likely to be suffering from moderate to severe depression as compared to married respondents (27%) (p<0.01). Unemployed individuals (40%) were more likely to be moderately to severely depressed as compared to employed (31%). Respondents with higher income and higher educational levels were less likely to be suffering from depression (Table 2). 

Increasing age increased the frequency of having OCD as depression was more frequent in the age group 55 years or older (81.2%) as compared to the age group 18-24 years old (48.1%) (p<0.01). Males showed more frequency of OCD (67.4%) than females (59.2%) (p<0.01). Moreover, married individuals and higher income groups showed a higher incidence of OCD. Higher education levels and employed individuals were more likely to have OCD (Table 3). 

## Discussion

Correlations have been drawn in the literature between previous pandemics and increased psychological distress [21]. We therefore have attempted to assess the frequencies of depression and OCD in the general population of Saudi Arabia during the COVID-19 pandemic.

The results showed that 65.2% of the respondents had depression and that females are more likely to develop moderate to severe depression during COVID-19 than males. This result agrees with a study conducted in China, with females showing higher psychological distress than males [6]. In addition, local studies have shown that females were at higher risk of developing psychological distress and depressive symptoms [21]. The high risk among females could be due to gender-related hormonal differences in response to stress [21]. Contrary to our findings, a study in China concluded no statistically significant difference between genders among the general Chinese population regarding depression associated with the COVID-19 pandemic [22]. Our results also showed that married individuals are less likely to suffer from severe depression than single individuals. In contrast, Lades et al. (2020) have reported less emotional wellbeing among married individuals, which could be associated with adjusting household difficulties during the quarantine [23]. Across age groups, our study indicates that the older population is less likely to develop depression compared to the younger population. Related evidence was reported from China as younger participants (<35 years) were more likely to develop depression during the COVID-19 pandemic than their older counterparts (≥35 years). A Chinese student living in Saudi Arabia committed suicide after being quarantined due to suspicions of being infected by COVID-19 virus. However, the first suicide case related to COVID-19 was reported in India in February 2020; a 50-year-old man misdiagnosed with COVID-19 committed suicide after reading mortality rates in the media [24]. Younger people spend more time on social media, which can mislead and influence their knowledge and judgement by unreliable information regarding COVID-19, causing significant panic and depression [21]. Social media is associated with negative effect on emotional wellbeing [23]. Also, the younger population is generally more involved in outdoor activities than the older age group [21], which was documented to enhance emotional wellbeing [23]. Thus, as a result of strict quarantine and lockdown measurements, daily activities have been dramatically curtailed [25]. Those reasons can justify our results of the highest frequency of severe depression among the youngest age group 18-24 years. During the pandemic, high unemployment rate was notable, which is associated with many unfavorable psychological outcomes, including increased anxiety and fear about future opportunities and career options [26]. Our study showed that individuals with lower socioeconomic status and those unemployed were more likely to be moderately to severely depressed. Furthermore, the economic downturn in certain countries led to widespread unemployment, which threatened workers' job security across different sectors, leading to mental distress [26]. When comparing educational levels, we observed that respondents with higher education are less likely to be depressed. While no statistically significant correlation was found in our study between depression and different nationalities, a study in China revealed that migrant workers had higher levels of distress [6].

Regarding OCD, our results showed that 62.4% most likely had OCD. Although OCD causes remain unexplained, there is a high prevalence among both genders. However, males are more likely to develop OCD during COVID-19 than females. Likewise, in a recent Canadian study, males were found to have more obsessive and compulsive symptoms [27]. We also found that married participants are most likely to have OCD. Another study had similar results in which single people were less likely to have obsessive symptoms [27]. Our results show that the older population is more likely to develop OCD. Additionally, our study found that OCD was most likely to occur as age increased, which is in concordance with the study conducted in Canada (p<0.01) [27]. The elderly population is at higher risk of developing complications [28]. Thus, they are more likely to adhere to the public health recommendations such as repeatedly washing hands to avoid the complications. Additionally, our study showed that employed people were more likely to suffer from OCD, which was also reported by the Canadian study [27]. However, respondents with high educational levels are more likely to develop OCD than those with lower education levels, as noted significantly in other studies [27]. No statistically significant correlation was found between OCD and different nationalities. However, Caucasians were more likely to suffer from obsessive and compulsive symptoms than Asians and other ethnicities in Canada [27]. The absence of associations in our study might be due to the small number of non-Saudi participants.

Our findings highlight that public health officials must increase their focus on raising awareness about mental health, especially during a pandemic. Increasing the awareness will help in limiting the stigma around this issue [29]. Some studies have shown that physical activity has positive effects for those with depression and OCD so it is important to highlight the importance of being physically active among those who suffer from depression and OCD [30]. 

One limitation of this study is selection bias, since only participants with access to social media platforms were able to participate. Also, this is a cross-sectional study, which means no causal relationship could be established. The questionnaire was self-administered, which may have caused recall bias. The response rate was higher than expected and it could be due to lockdown measures. The questions in the survey do not provide actual diagnosis of depression or OCD, it only provides initial assessment. However, since coronavirus is recent, this study adds a lot of value to the current literature since no studies have focused on both depression and OCD amid COVID-19 among the Saudi population, which eliminates publication bias. Future studies are needed to better understand mental health during lockdown. 

## Conclusions

The COVID-19 pandemic period is associated with significant mental health risks among the Saudi population. Females, younger population, single individuals, low socioeconomics, and low education level groups were found to be more likely to develop depression. Furthermore, males, older population, married individuals, low socioeconomics, unemployed, and high education level groups were at a higher risk for developing OCD. The results can provide further scientific support to better understand the impact of quarantine on psychological distress and mental health during emergency and disaster situations. 
